# Naringin Administration Is Associated with Reduced Reproductive Toxicity Following Subchronic Low-Dose Chlorpyrifos Exposure in Rats

**DOI:** 10.5812/ijpr-169653

**Published:** 2026-08-03

**Authors:** Solale Ramzani, Seyed Bagher Mortazavi, Mohammad Shokrzade, Ali Khavanin

**Affiliations:** 1 Department of Occupational Health and Safety Engineering, Faculty of Medical Sciences, Tarbiat Modares University, Tehran, Iran; 2 Department of Pharmacology and Toxicology, Faculty of Pharmacy, Mazandaran University of Medical Sciences, Sari, Iran

**Keywords:** Chlorpyrifos, Naringin, Male Infertility, Oxidative Stress

## Abstract

**Background:**

Male infertility is increasingly associated with environmental and occupational exposure to organophosphate pesticides, particularly chlorpyrifos (CPF). Chlorpyrifos persists in food, water, and the environment, resulting in chronic low-dose exposure. Although the toxic effects of high-dose exposure are well documented, the reproductive consequences of subchronic low-dose exposure remain unclear.

**Objectives:**

To evaluate whether intraperitoneal administration of naringin attenuates CPF-induced reproductive toxicity in adult male rats by assessing sperm parameters, serum reproductive hormones, oxidative stress markers, and testicular histopathology.

**Methods:**

Adult male rats were assigned to six groups (n = 5 per group): Control, naringin control (200 mg/kg), CPF (8 mg/kg), and CPF plus naringin (50, 100, or 200 mg/kg). Sperm count, motility, and morphology; serum testosterone, luteinizing hormone, and follicle-stimulating hormone levels; testicular malondialdehyde, reactive oxygen species, glutathione, and superoxide dismutase levels; and histopathological changes were assessed.

**Results:**

Chlorpyrifos exposure was associated with impaired sperm count, motility, and morphology; reduced reproductive hormone levels; increased oxidative stress; and testicular histological damage. Naringin pretreatment was associated with dose-dependent improvements in sperm parameters, partial restoration of hormonal balance, improvement in oxidative stress markers, and preservation of testicular histology, with more pronounced effects at 100 and 200 mg/kg.

**Conclusions:**

Naringin administration was associated with attenuation of CPF-induced reproductive alterations and improved oxidative stress markers and hormonal parameters. These findings support further investigation of naringin for pesticide-induced male reproductive dysfunction; however, validation in humans is required. The limited range of naringin doses and the absence of molecular investigations highlight the need for further studies to elucidate the underlying mechanisms and translational relevance to human reproductive health.

## 1. Background

Male infertility is a major global health concern and accounts for approximately half of all infertility cases worldwide. In recent decades, declining male reproductive health has been reported and has been largely attributed to environmental and occupational factors, particularly exposure to chemical pollutants such as pesticides (1). These exposures pose substantial risks because of their widespread use and persistence in the environment.

Organophosphorus pesticides are among the most widely used insecticides in agriculture and public health. Chlorpyrifos (CPF) [O,O-diethyl O-(3,5,6-trichloro-2-pyridyl) phosphorothionate] is a broad-spectrum organophosphate insecticide that remains widely used in many countries. Humans may be exposed to CPF through contaminated food and water and via inhalation and dermal contact, particularly in occupational settings (2). In addition to its well-known neurotoxic effects, increasing evidence indicates that CPF adversely affects the male reproductive system. Experimental studies have shown that CPF exposure impairs spermatogenesis, reduces sperm count and motility, increases sperm morphological abnormalities, and induces histopathological alterations in testicular tissue. Chlorpyrifos may also disrupt the hypothalamic-pituitary-gonadal axis, leading to reduced serum testosterone, luteinizing hormone (LH), and follicle-stimulating hormone (FSH) levels and indicating endocrine-disrupting effects (3).

Oxidative stress is considered a primary mechanism underlying CPF-induced reproductive toxicity. Chlorpyrifos exposure promotes excessive production of reactive oxygen species (ROS), leading to lipid peroxidation, mitochondrial dysfunction, DNA damage, and apoptosis in testicular cells. It also suppresses endogenous antioxidant defenses and interferes with genes involved in steroidogenesis and spermatogenesis. Epigenetic modifications, including altered DNA methylation patterns, have also been proposed to contribute to CPF-induced testicular toxicity (4, 5). Spermatozoa are particularly vulnerable to oxidative damage because of their high polyunsaturated fatty acid content and limited antioxidant capacity. Excessive oxidative stress can impair sperm membrane integrity, reduce motility, induce DNA fragmentation, and ultimately compromise male fertility. Consequently, antioxidants have attracted considerable attention as a potential strategy to protect against oxidative stress-mediated reproductive damage and improve male reproductive outcomes (6).

Flavonoids are naturally occurring polyphenolic compounds with well-documented antioxidant, anti-inflammatory, and cytoprotective properties (7). Naringin, a citrus flavonoid commonly found in grapefruit and other citrus fruits, exhibits strong antioxidant and free radical-scavenging effects (8). Previous studies suggest that naringin may improve redox balance and protect testicular tissue against toxic insults; however, evidence regarding its effects on male reproductive hormones and CPF-induced reproductive toxicity remains limited (9, 10).

## 2. Objectives

Given the widespread exposure to CPF and the vulnerability of the male reproductive system to oxidative stress, this study aimed to evaluate the potential protective effects of naringin against subchronic low-dose CPF-induced reproductive toxicity in male rats, with a particular emphasis on oxidative stress markers, reproductive hormones, sperm parameters, and testicular histopathological changes.

## 3. Methods

### 3.1. Reagents and Dose Administration

Technical-grade chlorpyrifos (70%) was obtained from Golsam Chemical Company (Iran). Chlorpyrifos was administered orally at 8 mg/kg, a sublethal dose based on previous toxicological studies and well below the oral LD_50_ in rats. In this study, “subchronic low-dose” refers to oral CPF administration at 8 mg/kg/day for 30 days (approximately 1/10 of the LD_50_), a regimen commonly used in experimental toxicology to investigate repeated CPF exposure (3). The experiment was conducted from November 5, 2025, to December 4, 2025.

Naringin (≥ 95% purity; Sigma-Aldrich, St Louis, Missouri, USA) was administered intraperitoneally at 50, 100, or 200 mg/kg. The 50- and 100-mg/kg doses were selected based on safety and efficacy data (11, 12), whereas the 200-mg/kg dose was used to assess dose-dependent effects. “Pretreatment” refers to daily intraperitoneal naringin administration beginning 3 days before CPF exposure and continuing throughout the experiment. Intraperitoneal administration was used to ensure accurate and reproducible systemic delivery and to minimize variability in absorption and first-pass metabolism (13).

### 3.2. Experimental Groups and Treatments

Adult male Wistar rats aged 10 to 12 weeks and weighing 220 to 250 g were obtained from the Animal Care Facility of Mazandaran University of Medical Sciences, Sari, Iran. The animals were housed under standard conditions (12-hour light/dark cycle, 21 ± 2 °C, and approximately 50% humidity) with free access to food and water. All procedures complied with the ARRIVE guidelines and were approved by the Institutional Ethics Committee (Approval No. IR.MODARES.AEC.1403.022). The rats were acclimatized to laboratory conditions for 7 days before the experiment. All animals were experimentally naive and were housed in groups of 3 to 4 per cage with wood shavings, bedding, and nesting material for enrichment. Health status was monitored daily, and only clinically healthy rats were enrolled. Predefined exclusion criteria included body weight loss greater than 20%, inability to eat or drink for more than 24 hours, severe lethargy or impaired mobility, or signs of sustained distress. No animals met these criteria; therefore, no exclusions were applied.

The rats were randomly assigned by simple randomization to 6 groups (n = 5 per group): Control, naringin control (200 mg/kg), CPF (8 mg/kg), and CPF plus naringin at 50, 100, or 200 mg/kg. Group size was determined based on ethical considerations and institutional approval to provide adequate statistical power to detect biologically relevant effects. Hormonal, biochemical, and sperm analyses were performed on coded samples by investigators blinded to group allocation. Histological evaluation was performed by a pathologist blinded to the experimental groups. Allocation concealment was not implemented because of the nature of daily treatment administration. All 30 animals were included in all end-point analyses. Treatments were administered once daily between 11:00 AM and 1:00 PM. Body weight was recorded weekly, and the animals were monitored for signs of toxicity or distress.

### 3.3. Blood and Tissue Collection

After 30 days, the rats were euthanized under light anesthesia with ketamine (40 mg/kg) and xylazine (5 mg/kg). Blood was collected and centrifuged, and serum was stored at −80 °C. The testes were excised; the right testis was fixed in 10% neutral buffered formalin for histopathological examination, and the left testis was homogenized for biochemical analyses, with the supernatants stored at −80 °C.

### 3.4. Serum Hormone Measurements

Serum testosterone, LH, and FSH were measured using commercial enzyme-linked immunosorbent assay kits (DRG Instruments GmbH, Marburg, Germany) according to the manufacturer’s instructions. Concentrations were expressed as ng/mL for testosterone and mIU/mL for LH and FSH.

### 3.5. Sperm Analysis

**Figure 1.**
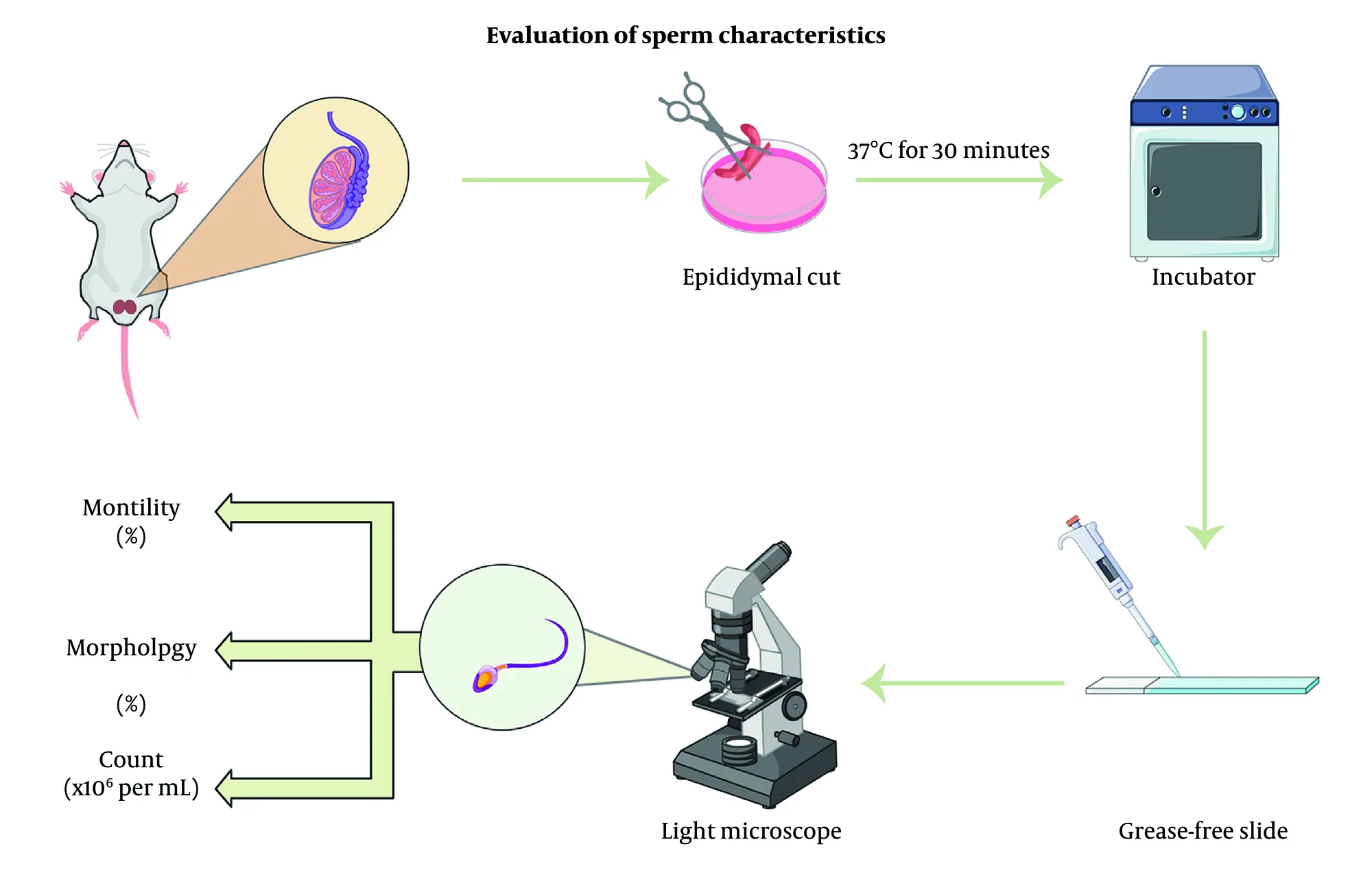
Evaluation of sperm characteristics

The sperm collection and evaluation procedure is shown in Figure 1. The cauda epididymis was excised and incubated in Dulbecco modified Eagle medium/F12 containing 10% fetal bovine serum at 37 °C for 30 minutes to release sperm. Sperm count, motility, and morphology were assessed in 200 spermatozoa per animal. Sperm count was determined manually using a Neubauer hemocytometer at ×400 magnification under a light microscope. Motility was evaluated in 10 microscopic fields, and morphology was assessed using eosin-nigrosin-stained smears according to established criteria (14). Sperm motility was expressed as the percentage of motile and nonmotile spermatozoa. Morphological abnormalities included amorphous heads, detached hooks, double heads, coiled tails, and other atypical forms. Mean values were calculated for each animal before statistical analysis. All sperm evaluations were performed by a single trained investigator blinded to group allocation.

### 3.6. Assessment of Oxidative Stress Markers

Testes were homogenized in mannitol buffer (pH 7.4) and centrifuged at 10,000 × g for 15 minutes at 4 °C. After protein quantification using the Bradford method (15), the supernatants were used for oxidative stress assays. Lipid peroxidation was measured as malondialdehyde (MDA) using the thiobarbituric acid assay (16), and results were expressed as nmol/g protein. Reactive oxygen species were quantified using the fluorescent probe 2′,7′-dichlorodihydrofluorescein diacetate, and fluorescence at 485/525 nm was normalized to protein content (17). Superoxide dismutase (SOD) activity was measured using a commercial colorimetric kit (Kushan Zist, Iran) based on inhibition of a chromogenic reaction, with absorbance measured at 450 nm (18). Glutathione (GSH) levels were measured using the 5,5′-dithiobis-(2-nitrobenzoic acid) method at 412 nm (19).

### 3.7. Histopathological Examination

Testicular tissues were fixed in formalin, processed by standard paraffin embedding, sectioned at 5 µm, and stained with hematoxylin and eosin (20). Histological assessment was performed under a light microscope (Olympus, Tokyo, Japan) by a blinded evaluator to assess seminiferous tubule morphology and testicular architecture. Spermatogenesis and tubular damage were quantified using Johnsen’s scoring system in 20 randomly selected seminiferous tubule cross-sections per animal (score range, 1 - 10). The mean score per rat was used for statistical analysis, and representative images were selected to illustrate group-specific histopathological changes.

### 3.8. Statistical Analysis

Data are presented as mean ± SD. One-way analysis of variance followed by the Tukey post hoc test was used for multiple comparisons among groups in SPSS version 25. All data met the assumptions of normality and homogeneity of variance. Each animal was considered an independent experimental unit. No animals or samples were excluded from the analyses.

## 4. Results

### 4.1. Sperm Characteristics

The treatment effects on sperm parameters are summarized in Table 1. Chlorpyrifos significantly reduced sperm count and motility (both P < 0.001) and increased morphological abnormalities (P = 0.004) compared with the control group, whereas naringin alone at 200 mg/kg had no adverse effects. Naringin pretreatment was associated with dose-dependent improvements in sperm parameters. Compared with the CPF group, naringin at 100 and 200 mg/kg significantly improved sperm count (P = 0.007 and P = 0.002, respectively) and motility (P = 0.011 and P = 0.004, respectively), although values remained below those in the control group.

**Table 1.** Sperm Characteristics in Adult Male Wistar Rats Following Subchronic Chlorpyrifos Exposure and Naringin Treatment ^a, b^

Sperm characteristic	Control	NRG 200	Chlorpyrifos	NRG 50 + CPF	NRG 100 + CPF	NRG 200 + CPF
**Count (× 10^6^/mL)**	64.24 ± 3.77	62.08 ± 3.08	29.76 ± 3.56 ^A, B^	41.12 ± 6.17 ^A, B, C^	46.83 ± 6.41 ^A, B, C^	61.52 ± 5.77 ^A, B, C^
**Motility (%)**	91.14 ± 6.91	82.15 ± 7.50	15.45 ± 4.76 ^A, B, C^	38.82 ± 4.09 ^A, B, C^	39.03 ± 6.71 ^A, B, C^	65.48 ± 8.75 ^A, B, C^
**Abnormality (%)**	2.74 ± 2.51	2.80 ± 2.41	34.34 ± 7.43 ^A, B^	29.55 ± 4.69 ^A, B^	30.43 ± 2.82 ^A, B^	32.66 ± 4.11 ^A, B^

^a^ Values are expressed as mean ± SD. Abbreviations: CPF, chlorpyrifos; NRG, naringin.

^b^ Different capital superscripted letters indicate significant differences within the same row (P ≤ 0.05): A, vs control; B, vs NRG 200; C, vs Diazinon; and D, vs chlorpyrifos.

### 4.2. Reproductive Hormone Levels

Changes in serum reproductive hormone levels are shown in Table 2. Chlorpyrifos exposure significantly reduced serum testosterone (P = 0.001), LH (P < 0.001), and FSH (P = 0.002) levels compared with the control group. Naringin pretreatment at 100 and 200 mg/kg significantly increased testosterone (P = 0.018 and P = 0.003, respectively) and LH (P = 0.029 and P = 0.012, respectively) levels compared with the CPF group. FSH levels improved significantly only at 200 mg/kg (P = 0.048) and did not fully return to control levels.

**Table 2.** Effects of Chlorpyrifos Exposure and Naringin Treatment on Serum Reproductive Hormones in Rats ^a, b^

Reproductive Hormone	Control	NRG 200	Chlorpyrifos	NRG 50 + CPF	NRG 100 + CPF	NRG 200 + CPF
**Testosterone (ng/mL)**	3.46 ± 0.05	3.40 ± 0.04	1.19 ± 0.03 ^A, B^	1.27 ± 0.02 ^A, B^	1.42 ± 0.03 ^A, B, C^	2.80 ± 0.05 ^A, B, C^
**LH (mIU/mL)**	7.47 ± 0.06	7.27 ± 0.03	3.49 ± 0.03 ^A, B^	3.39 ± 0.08 ^A, B^	3.77 ± 0.04 ^A, B, C^	4.10 ± 0.02 ^A, B, C^
**FSH (mIU/mL)**	5.52 ± 0.09	5.48 ± 0.02	2.56 ± 0.10 ^A, B^	2.42 ± 0.20 ^A, B^	2.86 ± 0.04 ^A, B^	3.04 ± 0.08 ^A, B, C^

^a^ Values are expressed as mean ± SD. Abbreviations: CPF, chlorpyrifos; FSH, follicle-stimulating hormone; LH, luteinizing hormone; NRG, naringin.

^b^ Different capital superscripted letters indicate significant differences within the same row (P ≤ 0.05): A, vs control; B, vs NRG 200; C, vs Diazinon; and D, vs chlorpyrifos.

### 4.3. Oxidative Stress Markers

**Figure 2.**
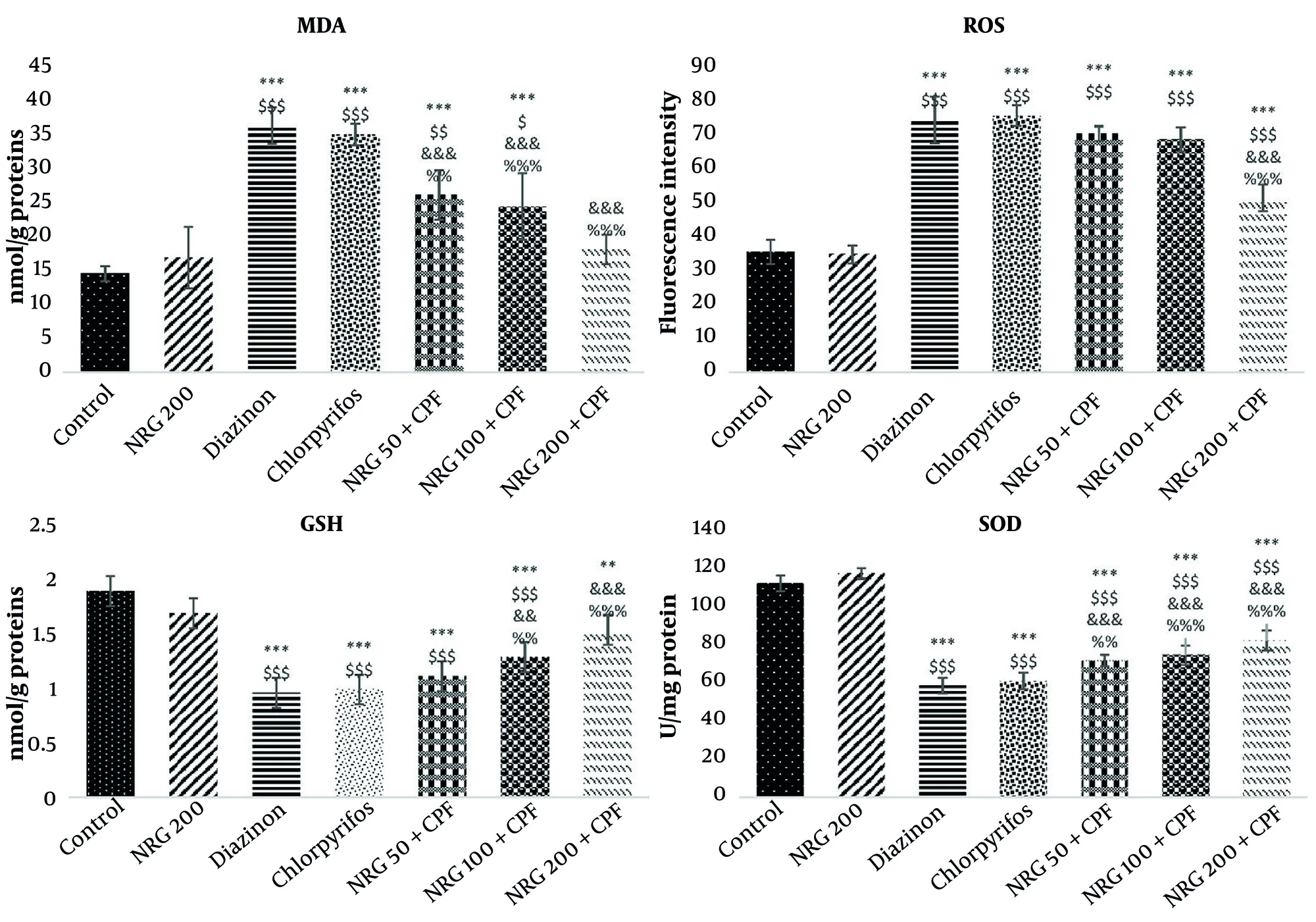
Reactive oxygen species (ROS), glutathione (GSH), malondialdehyde (MDA), and superoxide dismutase (SOD) levels in testicular tissues across experimental groups. Control, untreated animals; NRG 200, rats receiving naringin alone (200 mg/kg); chlorpyrifos (CPF), rats receiving chlorpyrifos; NRG 50 + CPF, rats receiving naringin 50 mg/kg plus chlorpyrifos; NRG 100 + CPF, rats receiving naringin 100 mg/kg plus chlorpyrifos; and NRG 200 + CPF, rats receiving naringin 200 mg/kg plus chlorpyrifos. Values are presented as mean ± SD (n = 5). Statistical symbols indicate *P < 0.05, **P < 0.01, and ***P < 0.001 vs control; $P < 0.05, $$P < 0.01, and $$$P < 0.001 vs NRG 200; and %P < 0.05, %%P < 0.01, and %%%P < 0.001 vs chlorpyrifos.

As shown in Figure 2, CPF significantly increased MDA and ROS levels and reduced GSH levels and SOD activity compared with the control group (all P < 0.001). Naringin pretreatment was associated with dose-dependent improvements in oxidative stress markers, with the most pronounced effects at 200 mg/kg. However, values did not fully return to control levels.

### 4.4. Histopathological Findings

**Figure 3.**
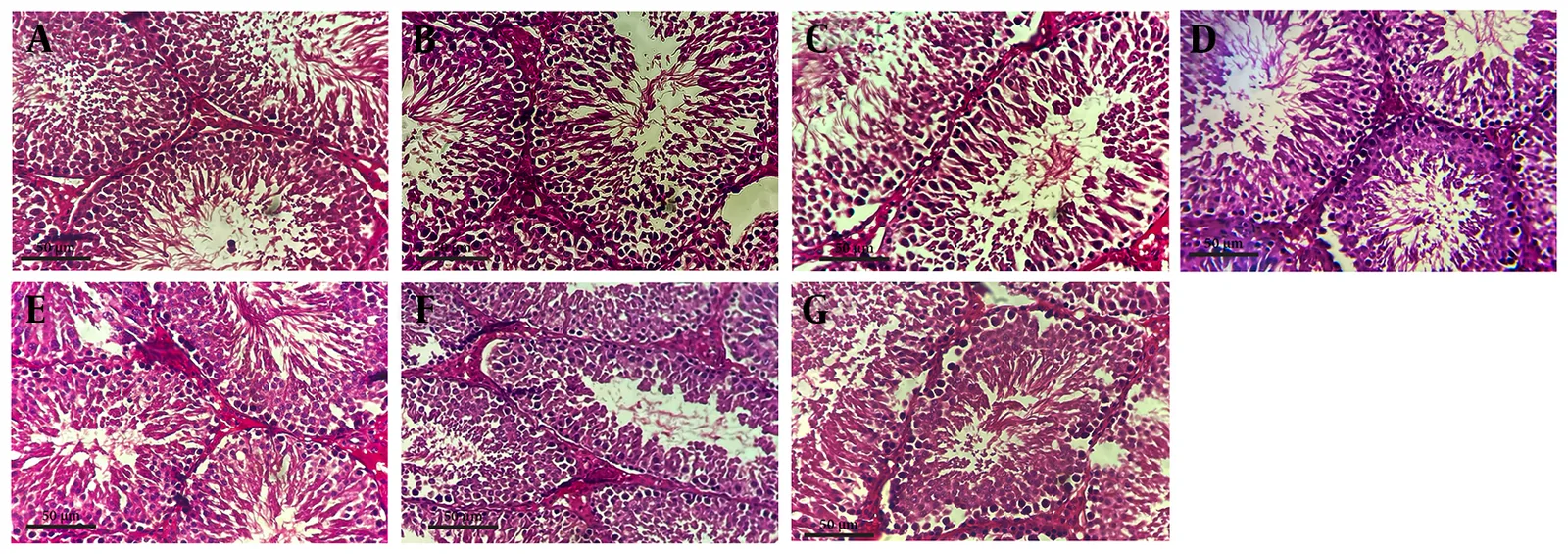
Histological architecture of rat testes across experimental groups. Representative hematoxylin and eosin-stained testicular sections from the control (A), naringin 200 mg/kg (B), CPF (C), naringin 50 mg/kg + CPF (D), naringin 100 mg/kg + CPF (E), and naringin 200 mg/kg + CPF (F) groups. The control and naringin 200-mg/kg groups showed normal seminiferous tubules, orderly germ cells, and intact Sertoli and Leydig cells. Chlorpyrifos induced severe seminiferous epithelial disorganization, vacuolation, germ cell exfoliation, necrosis, and interstitial edema. Naringin 50 mg/kg + CPF produced minimal improvement; 100 mg/kg + CPF partially restored tubular structure and spermatogenic layers, with focal Sertoli cell depletion; and 200 mg/kg + CPF markedly preserved tubular architecture and spermatogenesis, with mild residual vacuolation. Scale bar = 50 µm.

Histopathological findings are shown in Figure 3. The Johnsen scores were 9.82 ± 0.15 and 9.74 ± 0.22 in the control and naringin-only groups, respectively, indicating normal testicular architecture. Chlorpyrifos exposure caused severe histopathological alterations, including epithelial disorganization, germ cell loss, Sertoli cell depletion, and interstitial edema, and significantly reduced the Johnsen score to 3.22 ± 0.51 (P < 0.001 vs control). Naringin pretreatment attenuated CPF-induced testicular damage. Naringin at 50 mg/kg had no significant effect (3.44 ± 0.48), 100 mg/kg partially restored testicular structure (5.68 ± 0.64; P < 0.01 vs CPF), and 200 mg/kg markedly improved testicular morphology and increased the Johnsen score to 8.26 ± 0.72 (P < 0.001 vs CPF).

## 5. Discussion

Subchronic low-dose CPF exposure was associated with substantial male reproductive toxicity in rats, including impaired sperm parameters, disrupted reproductive hormone levels, testicular oxidative stress, and severe histopathological damage. Naringin pretreatment was associated with dose-dependent attenuation of these CPF-induced alterations.

Chlorpyrifos markedly reduced sperm count and motility and increased morphological abnormalities, possibly through disruption of gonadotropin and androgen levels and induction of testicular oxidative damage (4, 21, 22). Reduced LH and testosterone levels can impair spermatogenesis, whereas oxidative stress compromises mitochondrial function, adenosine triphosphate production, and sperm viability. Naringin was associated with dose-dependent improvements in CPF-induced sperm toxicity. Higher doses (100 - 200 mg/kg) significantly improved sperm parameters and partially reduced abnormalities, possibly through preservation of Leydig cell function and reduction of ROS-mediated sperm damage (23). Naringin alone at 200 mg/kg did not impair spermatogenesis.

Male rats exposed to CPF also showed significant reductions in serum testosterone, LH, and FSH levels, indicating disruption of reproductive hormone balance. These changes may be associated with reduced pituitary secretion, altered testosterone metabolism, and downregulation of genes involved in gonadotropin synthesis and steroidogenesis (24). However, these molecular pathways were not directly assessed in this study and should be considered potential mechanisms that require further investigation.

Oxidative stress and degenerative effects on Leydig cells further impair steroidogenesis and spermatogenesis (25). Naringin partially restored reproductive hormone levels, particularly at 200 mg/kg, consistent with previous studies reporting attenuation of endocrine toxicity (23).

Chlorpyrifos disrupted the testicular oxidant-antioxidant balance, as shown by reduced GSH levels and SOD activity and increased MDA and ROS levels. These changes are consistent with oxidative stress and may involve mitochondrial dysfunction, lipid peroxidation, and reduced antioxidant enzyme activity (26-28). Although some studies have reported increased testicular antioxidant activity after CPF exposure, these discrepancies may reflect dose- and duration-dependent adaptive responses (25). Naringin was associated with dose-dependent improvements in antioxidant status, reduced ROS levels, and preservation of testicular function, potentially because of its reported free radical-scavenging, metal-chelating, and anti-inflammatory properties (29).

Chlorpyrifos exposure caused marked histopathological alterations, including disorganization of the germinal epithelium, Sertoli cell depletion, germ cell apoptosis, and interstitial edema, indicating severe testicular toxicity. These findings are consistent with previous reports of organophosphate-induced testicular damage (22). Naringin pretreatment preserved testicular architecture in a dose-dependent manner, with the greatest preservation at 200 mg/kg. The higher Johnsen scores supported preservation of seminiferous tubule structure and spermatogenic activity (23, 30).

### 5.1. Study Limitations

This rodent study has several limitations. The range of naringin doses was limited, molecular and epigenetic pathways were not assessed, and long-term outcomes were not evaluated. Extrapolation to humans should therefore be cautious because of species differences and limited mechanistic evidence. Future studies using human models and epidemiological approaches are needed to clarify the mechanisms and translational relevance of these findings.

### 5.2. Conclusions

Naringin administration was associated with attenuation of CPF-induced male reproductive toxicity in rats, accompanied by improvements in sperm quality, hormonal balance, oxidative stress markers, and testicular structure. These findings suggest that naringin may have potential as a natural agent against pesticide-induced reproductive disorders; however, confirmation in long-term studies and human populations is required. The observed effects were associated with changes in oxidative stress and hormonal parameters rather than direct mechanistic evidence. Future studies should examine broader dose ranges, longer treatment durations, and underlying molecular mechanisms to define the potential protective role of naringin more clearly.

## Data Availability

The dataset presented in the study is available on request from the corresponding author during submission or after publication.
